# Endocrine treatment mechanisms in triple-positive breast cancer: from targeted therapies to advances in precision medicine

**DOI:** 10.3389/fonc.2024.1467033

**Published:** 2025-01-07

**Authors:** Xiu Yang, Daxin Yang, Xue Qi, Xiujuan Luo, Guangmei Zhang

**Affiliations:** Department of Medical Oncology, Third Division, Jilin City Second People’s Hospital, Jilin, China

**Keywords:** triple-positive breast cancer, molecular mechanisms, endocrine therapy resistance, precision medicine, gene editing (CRISPR/Cas9)

## Abstract

Triple-positive breast cancer (TPBC), defined by the co-expression of estrogen receptor (ER), progesterone receptor (PR), and human epidermal growth factor receptor 2 (HER2), poses unique therapeutic challenges due to complex signaling interactions and resulting treatment resistance. This review summarizes key findings on the molecular mechanisms and cross-talk among ER, PR, and HER2 pathways, which drive tumor proliferation and resistance to conventional therapies. Current strategies in TPBC treatment, including endocrine and HER2-targeted therapies, are explored alongside emerging approaches such as immunotherapy and CRISPR/Cas9 gene editing. Additionally, we discuss the tumor microenvironment (TME) and its role in treatment resistance, highlighting promising avenues for intervention through combination therapies and predictive biomarkers. By addressing these interdependent pathways and optimizing therapeutic strategies, precision medicine holds significant potential for improving TPBC patient outcomes and advancing individualized cancer care.

## Introduction

Breast cancer remains a global health challenge, significantly impacting women’s well-being worldwide. As the most prevalent cancer among women, it accounts for over 2 million new cases and approximately 600,000 deaths annually, highlighting the pressing need for effective treatment and management strategies (1–3). Although breast cancer primarily affects women, it also occurs in men, albeit rarely, accounting for about 1% of cases. This fact underscores the importance of understanding breast cancer across all genders (4). Disparities in breast cancer outcomes are particularly evident between developed and developing regions; developed regions benefit from early detection, public awareness initiatives, and advanced treatment options, while developing regions often experience delayed diagnoses and lower survival rates (5, 6). Addressing these disparities requires tailored healthcare strategies that consider socioeconomic factors.

The 2011 St. Gallen Breast Cancer Consensus proposed a classification of breast cancer into four molecular subtypes based on estrogen receptor (ER), progesterone receptor (PR), human epidermal growth factor receptor 2 (HER2), and Ki-67 proliferation index. These subtypes are luminal A (ER and/or PR positive, HER2 negative, low Ki-67 expression), luminal B (ER and/or PR positive, HER2 negative with high Ki-67 or HER2-positive), HER2-overexpressed (ER and PR negative, HER2 positive), and basal-like (ER and PR negative, HER2 negative) (7). Among these, the luminal B subtype with HER2 positivity—where ER and PR are positive, and HER2 is amplified or overexpressed—is known as triple-positive breast cancer (TPBC), representing approximately 10% to 15% of all breast cancers (8).

Triple-positive breast cancer is characterized by hormone dependency, where estrogen binding to receptors in tumor cells activates transcription of target genes, including those responsible for PR synthesis. Additionally, HER2 signaling plays a crucial role in driving tumor proliferation and resistance mechanisms, making TPBC distinctively challenging to treat. This receptor interplay is critical in regulating breast cell growth and differentiation, highlighting TPBC as a complex and unique form of breast cancer. However, managing TPBC is particularly challenging due to its intrinsic heterogeneity and variability in patient responses. The complexity of this disease—shaped by tumor biology, genetic influences, and the tumor microenvironment—complicates treatment strategies and frequently contributes to resistance against standard endocrine therapies (9, 10). These challenges underscore the need for a highly personalized treatment approach that integrates the latest advances in precision medicine and combination molecular targeting to enhance therapeutic efficacy and improve patient outcomes (11).

This review provides a foundational overview of the molecular mechanisms, signaling pathways, and clinical implications of endocrine therapy in TPBC. By synthesizing current research and recent discoveries, this article aims to clarify primary challenges and highlight innovative strategies that hold promise for transforming treatment approaches and enhancing patient care in this complex clinical context.

## Molecular mechanisms and signaling pathways in triple-positive breast cancer

Triple-positive breast cancer is uniquely defined by the concurrent expression of estrogen receptor (ER), progesterone receptor (PR), and human epidermal growth factor receptor 2 (HER2), each playing a distinct role in tumor growth and survival. ER and PR are key drivers of hormone-dependent growth, while HER2 amplifies proliferative and survival signals, often leading to aggressive tumor behavior. Understanding the molecular interactions between ER, PR, and HER2 is essential, as these signaling pathways collectively inform therapeutic strategies and contribute significantly to treatment resistance. This review first examines ER signaling, explores the interplay between PR and HER2, and concludes with a discussion on cross-regulation among these pathways, highlighting their role in fostering therapeutic resistance. This approach offers a comprehensive view of how these signaling networks shape TPBC’s distinct biological and clinical behavior, emphasizing the need for a precise, molecularly informed approach to treatment (12, 13).

### Structural and functional aspects of estrogen receptors

Estrogen receptors (ERs), particularly ERα and ERβ, are integral members of the steroid hormone nuclear receptor family and act as ligand-dependent transcriptional regulators. Each receptor contains five domains (A through F) with distinct functions. The N-terminal A/B domain houses the ligand-independent transcription activation function AF-1 (14). The central C domain features a DNA-binding region with zinc finger motifs that enable binding to estrogen response elements (ERE) and promote receptor dimerization (15). The D domain serves as a flexible hinge, and the C-terminal E domain functions as the ligand-binding region, which distinguishes estrogen activators from antagonists (16). Finally, the F domain contains AF-2, essential for ligand-dependent transcriptional activation (17).

Activation of ER generally involves ligand binding, which facilitates ERE interaction on target genes, driving essential transcription for cellular proliferation. Beyond these genomic effects, ER also engages in non-genomic actions through interactions with transcription factors and kinases, thus broadening its influence on cellular growth and the tumor microenvironment (18).

### Disruption of ER expression

ER expression can be impacted by methylation in regions upstream of the ESR1 gene itself, particularly at CpG islands, which can suppress ERα expression and affect its regulatory role in cellular growth (1). Additionally, loss of function in genes like Cyclin-dependent kinase 10 (CDK10) has been linked to endocrine resistance, as CDK10 deficiency may lead to altered cell cycle regulation and increased activation of mitogenic pathways, which can contribute to reduced sensitivity to estrogen signaling (19). This loss of ER function and associated resistance mechanisms highlight the significant implications of ER dysregulation in cancer progression.

### Classification and mechanism of the EGFR family

The Epidermal Growth Factor Receptor (EGFR) family belongs to the type I tyrosine kinase receptor superfamily and includes four closely related receptors: EGFR, HER2, HER3, and HER4. These receptors share highly homologous amino acid sequences and exhibit similar structural characteristics. When specific ligands, such as TGFα and EGF, bind to these receptors, they can form homodimers or heterodimers, activating intracellular tyrosine kinase domains. This activation triggers the autophosphorylation of tyrosine residues and initiates several critical downstream pathways, including Ras-Raf-MEK-MAPK, PI3K/AKT/mTOR, and Erk/MAPK. These pathways are essential for cell proliferation and are associated with adverse outcomes in breast cancer, such as tumor recurrence, chemotherapy resistance, and reduced sensitivity to endocrine therapies (20–22).

### HER2/neu and its role in breast cancer

HER2/neu, often referred to as HER2 or neu, is a proto-oncogene located on chromosome 17 that encodes the P185 phosphoprotein. When estrogen binds to its receptor (ER) on the nuclear membrane, it typically upregulates HER2 ligands and downregulates the receptor itself, activating the ER and subsequently phosphorylating multiple protein kinases involved in downstream signaling pathways. Once activated, ER can interact with nuclear coactivators like AIB1, significantly boosting HER2’s transcriptional activity. This ER-HER2 interaction enhances the tumor cells’ proliferative capacity, providing a distinct growth advantage that is crucial in the progression of breast cancer. This mechanism underscores the complex interplay between hormone signaling and receptor tyrosine kinase pathways, which has substantial implications for therapeutic strategies in breast cancer treatment (8, 20, 21).

### HER2 and ER interactions: genomic and non-genomic actions

The interaction between HER2 and ER signaling pathways is key to understanding the progression of triple-positive breast cancer. While each pathway has its role, their interactions result in a highly coordinated system that influences tumor growth and therapeutic resistance. Once ER is activated on the nuclear membrane, it regulates gene expression to drive breast cancer cell proliferation. HER2 signaling, however, can enhance ER-mediated effects, highlighting the intricate cross-talk between these pathways.

ER activity is modulated by co-regulatory proteins—classified into co-activators and co-inhibitors—that influence ER’s transcriptional effectiveness on estrogen response elements (ERE) in target genes. Co-activators increase ER’s transcriptional impact, while co-inhibitors decrease it. The balance between these co-regulators can shift, and excessive co-activator expression or suppressed co-inhibitors may diminish the efficacy of endocrine therapies such as SERMs (23).

### Amplified in breast cancer 1 and its role in resistance

Amplified In Breast Cancer 1 (AIB1), an ER co-activator, exists in both normal and some breast cancer tissues, activating essential signaling pathways like PI3K/Akt and ERK/MAPK that promote tumor growth. Research indicates that HER2 overexpression in ER-positive cell lines, such as MCF-7, amplifies proliferation signals significantly. When HER2 signaling upregulates AIB1, tamoxifen (TAM) transitions from acting as an antagonist to functioning as an agonist, thereby promoting tumor growth and contributing to resistance (12).

Further studies utilizing RNA interference to decrease endogenous AIB1 levels in lung and breast cancer cells reveal that reducing AIB1 expression diminishes the response to growth signals (24). This AIB1 reduction results in decreased phosphorylation of multiple HER2 tyrosine residues and lowers the activity of the co-regulatory protein Src. When HER2 is transfected into MCF-7 cells and transplanted into nude mice, estrogen presence significantly accelerates tumor growth. Removing estrogen causes tumor regression, suggesting that HER2 overexpression increases ER sensitivity to proliferation signals and reduces TAM effectiveness. High AIB1 levels can substantially reduce TAM’s antagonistic effects in HER2-overexpressing tumors, highlighting the intricate relationship between genomic and non-genomic pathways in breast cancer resistance.

Moreover, the activation of tyrosine kinase receptors, such as EGFR, can increase ER phosphorylation at serine 118, reducing estrogen receptor expression and elevating kinase levels in downstream signaling pathways like ERK/MAPK, which further contributes to endocrine therapy resistance. SERMs can stimulate several signaling pathways through membrane-initiated steroid signaling that occurs outside the nucleus and independently of gene transcription. These signals, partly mediated by ER fragments near or on the endoplasmic reticulum, can reduce SERM efficacy and cause therapeutic failure (21). Long-term estrogen deprivation (LTED) can enhance non-genomic signaling pathways in breast cancer cells, potentially increasing ERα interactions at the cell membrane. This adaptation may involve phosphorylation of Shc protein upon estrogen binding, which can activate the HER2/Ras/Raf/MAPK signaling cascade, facilitating cell proliferation and transcriptional activity of nuclear factors (12).

### Interactions and therapeutic implications of ER, HER2, and PR in triple-positive breast cancer

Interactions among ER, PR, and HER2 in triple-positive breast cancer highlight the limitations of monotherapy approaches that target a single receptor. Reciprocal signaling between these receptors contributes to tumor aggressiveness and fosters resistance to targeted treatments. This interconnected signaling dynamic underscores the need for therapeutic strategies that simultaneously inhibit multiple pathways, which may help overcome resistance mechanisms and improve patient outcomes (8, 25). Furthermore, this complex interplay among ER, PR, and HER2 reinforces the importance of precision medicine in TPBC management. A thorough molecular characterization—including receptor status, signaling pathway activations, and relevant mutations—can inform tailored treatment strategies that address the tumor’s unique biological features (26, 27).

### Complexity of signal cross-regulation in triple-positive breast cancer

The aggressive phenotype of triple-positive breast cancer is heavily influenced by the intricate cross-regulation among ER, PR, and HER2. These inter-receptor dynamics not only drive tumor aggressiveness but also significantly impact response to targeted therapies. One example of this complexity is the synergistic effect between HER2 activation and estrogen-driven tumor growth: HER2 amplification can enhance estrogen’s proliferative effects, which may undermine traditional anti-estrogen therapies, as these treatments can inadvertently intensify HER2-mediated signaling. Conversely, ER activation can upregulate HER2 expression, establishing a positive feedback loop that accelerates tumor progression. Together, ER, PR, and HER2 regulate key cellular processes, such as cell cycle progression, DNA repair, adhesion, and migration, contributing to a highly invasive and metastatic tumor phenotype. This complexity presents substantial treatment challenges, highlighting the need for therapeutic strategies that concurrently target multiple pathways to effectively manage TPBC (9, 12).

### Therapeutic considerations for interactions among ER, PR, and HER2

A comprehensive understanding of the interactions between estrogen receptor (ER), progesterone receptor (PR), and human epidermal growth factor receptor 2 (HER2) is crucial for developing effective treatment strategies for triple-positive breast cancer. Studies have shown that combining endocrine therapy with HER2-targeted agents, such as lapatinib and trastuzumab, improves treatment outcomes in triple-positive breast cancer patients compared to endocrine therapy alone (28). This underscores the importance of multi-targeted therapeutic strategies in overcoming resistance and improving patient outcomes. Furthermore, the molecular interactions among ER, PR, and HER2—especially involving coactivators like AIB1 and decreased expression of corepressors—present potential intervention points. Targeting these critical nodes within the signaling network may suppress tumor growth and counteract resistance mechanisms, introducing innovative treatment strategies (9, 12).

Recent studies have also examined inhibiting kinases involved in the cross-talk between ER and HER2 signaling or targeting regulators of receptor activity as methods to overcome resistance (29, 30). These approaches highlight the potential of combining hormone therapy with inhibitors targeting HER2 and other key signaling elements. Such strategies aim to disrupt pathways that support tumor growth and adaptability, enhancing therapeutic effectiveness and improving patient outcomes (13, 27).

## Overview of therapeutic challenges and approaches in triple-positive breast cancer

Triple-positive breast cancer, characterized by the co-expression of estrogen receptor (ER), progesterone receptor (PR), and human epidermal growth factor receptor 2 (HER2), presents unique treatment challenges. The intricate cross-talk among these receptors frequently drives therapeutic resistance, complicating treatment outcomes. Addressing this interplay requires comprehensive strategies that effectively target these pathways.

### Endocrine therapy and resistance mechanisms

Endocrine therapy (ET) is fundamental for managing hormone-dependent, ER-positive triple-positive breast cancer by targeting estrogen signaling. Common treatments include selective estrogen receptor modulators (SERMs), like tamoxifen, and aromatase inhibitors (AIs), like letrozole, particularly for postmenopausal patients (31, 32). However, approximately 30% of patients treated with AIs experience disease progression due to alternative pathway activation, notably the PI3K/Akt/mTOR pathway (33). Resistance often emerges within the first five years of treatment and is associated with HER2-driven bypass mechanisms that circumvent ER-blocking therapies, such as tamoxifen, allowing tumor progression (9, 10, 31).

Combining ET with HER2-targeted therapies has shown improved outcomes. For instance, patients receiving a combination of endocrine and HER2-targeted therapy achieved a median overall survival (OS) of 56.5 months, compared to 38.9 months with endocrine therapy alone (34). This finding underscores the importance of combination therapies to effectively disrupt estrogen signaling and inhibit tumor growth and spread. In the MONARCH 2 trial, abemaciclib combined with fulvestrant yielded a PFS increase to 16.4 months compared to 9.3 months with fulvestrant alone, supporting CDK4/6 inhibitors’ use in managing hormone receptor-positive breast cancers. For TPBC, this indicates a potential strategy to mitigate resistance (27).

### Selective estrogen receptor modulators and downregulators

SERMs, such as tamoxifen and raloxifene, block ERs in breast tissue, thereby inhibiting tumor growth, while also acting as agonists in bone and cardiovascular tissues. However, long-term SERM use can lead to resistance and side effects like increased blood clot risks (31, 35). In contrast, selective estrogen receptor downregulators (SERDs), such as fulvestrant, provide a more comprehensive approach by degrading ER entirely, effectively blocking its cancer-promoting effects.

SERDs are particularly useful for patients who develop resistance to AIs or tamoxifen, and studies like the FALCON and FIRST trials have shown that fulvestrant extends progression-free survival (PFS), especially in patients without visceral metastasis (36, 37). Ongoing research is exploring the combined use of fulvestrant with CDK4/6 inhibitors, which could extend PFS further, though with potential risks like myelosuppression (38).

### LHRH antagonists in premenopausal women

For premenopausal women, luteinizing hormone-releasing hormone (LHRH) antagonists offer additional therapeutic benefits by lowering estrogen levels through ovarian suppression, thus reducing cancer proliferation. Studies indicate that combining LHRH agonists with tamoxifen yields superior results, improving both OS and PFS compared to LHRH agonist monotherapy (39). This combination is therefore recommended for premenopausal women eligible for endocrine therapy.

### HER2-targeted therapy: dual inhibition and combination approaches

HER2-targeted therapies, including trastuzumab and pertuzumab, have transformed the treatment landscape for HER2-positive and triple-positive breast cancers. When combined with chemotherapy or endocrine therapy, these therapies have significantly improved progression-free survival (PFS) and overall survival (OS) (26). However, resistance may develop due to mutations in downstream pathways or HER2 expression loss, and potential cardiotoxicity poses risks, particularly for patients with pre-existing cardiovascular conditions (22) (Moasser, 2007).

Dual HER2 Inhibition: Dual inhibition, combining trastuzumab with lapatinib, has demonstrated synergistic effects that surpass single-agent therapy. For instance, the NeoALTTO trial compared trastuzumab with lapatinib and found a higher pathological complete response (pCR) rate (51.3%) for dual therapy compared to trastuzumab alone (29.5%) (26). Although a follow-up study did not show a statistically significant improvement in 3-year event-free survival (EFS) over trastuzumab alone, pCR patients showed better survival outcomes. This underscores dual HER2 inhibition’s potential in improving neoadjuvant therapy results. Side effects, however, include elevated liver enzymes and diarrhea, highlighting the need for careful patient management (40). In advanced HER2-positive breast cancer, recent data from the KATE2 trial showed that adding atezolizumab to trastuzumab emtansine (T-DM1) extended progression-free survival (PFS) to 8.2 months compared to 6.8 months for T-DM1 alone, suggesting that immune modulation may benefit specific patient subsets (41)​.

### Immunotherapy: emerging role and challenges

Immune checkpoint inhibitors (ICIs) represent an emerging avenue in breast cancer treatment, showing promise particularly in triple-negative breast cancer (TNBC). Early trials are now exploring the potential of ICIs for triple-positive breast cancer, especially in combination with HER2-targeted therapies (42). While immunotherapy engages the immune system to target cancer cells, it also introduces immune-related adverse events (irAEs) such as autoimmune reactions. The long-term effectiveness and safety of ICIs in triple-positive breast cancer remain areas of active investigation (41). In the KEYNOTE-355 trial, 847 patients with advanced TNBC were randomized to receive either pembrolizumab plus chemotherapy or a placebo plus chemotherapy. Among patients with a PD-L1 CPS score ≥10, the pembrolizumab-chemotherapy group showed a median progression-free survival (PFS) of 9.7 months, compared to 5.6 months with placebo-chemotherapy (hazard ratio [HR], 0.65; 95% CI 0.49–0.86; one-sided p=0.0012), indicating a significant improvement in PFS. However, in the broader CPS ≥1 group, the PFS improvement was less pronounced (7.6 vs. 5.6 months; HR 0.74; one-sided p=0.0014, not statistically significant), and OS results did not show statistical significance (43).

### Gene therapy: CRISPR/Cas9 in triple-positive breast cancer treatment

CRISPR/Cas9 technology has become increasingly relevant in breast cancer research, offering potential solutions to drug resistance and potentially enhancing the efficacy of immunotherapy in CRISPR-targeted cancer therapies in the near future (44). This gene-editing approach allows precise targeting and modification of genes associated with treatment resistance and tumor growth, making it particularly valuable for difficult-to-treat subtypes like triple-positive breast cancer. One promising application of CRISPR/Cas9 is the modulation of protein degradation pathways within cancer cells to control tumor proliferation. The 26S proteasome, a multi-catalytic enzyme responsible for degrading polyubiquitinated proteins, regulates proteins involved in the cell cycle and apoptosis, such as caspases (44). Inhibiting proteasome function in cancer cells exhibits antitumor and pro-apoptotic effects by sensitizing cells to intrinsic and extrinsic apoptotic signals, making the proteasome an important target for cancer therapies. Moreover, site-specific phosphorylation of the proteasome has been linked to breast cancer proliferation, suggesting that disrupting this activity could aid in disease control (45). CRISPR/Cas9 has also been applied to knock out dual-specificity tyrosine-regulated kinase 2 (DYRK2), a kinase involved in proteasome regulation. This approach has been shown to inhibit tumor growth in proteasome-addicted human breast carcinoma models in mice (46). In HER2-positive breast cancers, CRISPR/Cas9 has been used to disrupt HER2 signaling pathways, which enhances the effectiveness of PARP inhibitors. Additionally, CRISPR/Cas9 targets metabolic genes like FASN—essential for fatty acid synthesis in cancer cells—as well as transcription factors such as FOXA1 and CDK7, providing new strategies to control aggressive breast cancer subtypes (47). This precision in gene editing supports the development of personalized treatment strategies, which can reduce the likelihood of resistance by addressing specific genetic alterations. In estrogen receptor (ER)-positive breast cancer, resistance to therapies like tamoxifen and aromatase inhibitors often arises from mutations in ERα, such as ERαY537S and ERαD538G, which contribute to more aggressive metastatic disease that is less responsive to standard treatments (48). To explore the impact of these mutations, CRISPR/Cas9 has been utilized to create ERα-positive breast cancer models where the wild-type ERα gene is replaced with the mutated forms ERαY537S or ERαD538G, allowing researchers to study resistance mechanisms and develop new therapeutic strategies. Additionally, CRISPR/Cas9 can be applied to downregulate MYC, a gene highly expressed in high-grade breast cancers, by editing its regulatory elements, which curbs cancer cell proliferation (49). Another notable target in breast cancer progression is migration and invasion enhancer 1 (MIEN1), a protein associated with tumor metastasis. Overexpression of MIEN1 promotes cancer cell migration and invasion, making it a critical target for limiting metastatic spread (48). CRISPR/Cas9 has been used to delete the MIEN1 gene in breast cancer models, delivered via cloning vectors such as [pSpCas9(BB)-2A-GFP (PX458)], which successfully silenced MIEN1 expression and inhibited disease progression (50). In summary, CRISPR/Cas9 gene editing holds significant potential for breast cancer treatment by allowing precise disruption of oncogenes and enhancement of tumor suppressor functions. This technology provides new avenues to tackle drug resistance, control tumor growth, and improve therapeutic outcomes in various breast cancer subtypes.

### Precision medicine in triple-positive breast cancer

Precision medicine has become essential in managing triple-positive breast cancer by tailoring treatment to individual characteristics to optimize outcomes. Key clinical factors—such as age, menopausal status, hormone receptor status, HER2 expression, and markers like Ki-67—guide the most suitable treatment strategy (26, 51). For example, younger premenopausal women with high Ki-67 levels may benefit from aggressive HER2-targeted therapy in combination with chemotherapy. Conversely, older postmenopausal women with low Ki-67 levels may respond well to endocrine therapy with a lower toxicity profile (25, 52). Patients with cardiovascular risks should be closely monitored when receiving HER2-targeted therapy due to cardiotoxicity. In such cases, less cardiotoxic agents or endocrine-only therapies may be preferable (52, 53). Additionally, patients with autoimmune histories may face higher risks from immunotherapy due to potential irAEs, underscoring the need for careful patient selection and monitoring (42).

### Clinical use and strategic considerations

For effective management of triple-positive breast cancer (TPBC), endocrine therapy must be carefully tailored to the unique biological characteristics of each tumor. Key decision factors include hormone receptor status, HER2 expression, and proliferation markers like Ki-67. High levels of ER and PR often indicate a strong reliance on hormonal signaling, suggesting that selective estrogen receptor modulators (SERMs) or aromatase inhibitors (AIs) could be particularly effective. In contrast, tumors with HER2 amplification require a comprehensive approach that includes HER2-targeted therapies to optimize patient outcomes (25, 51).

## Comprehensive approach to evaluating therapy effectiveness

To fully assess hormone therapy (HT) effectiveness in TPBC, an integrative approach encompassing imaging, biomarker analysis, and quality-of-life evaluation is essential. This multidimensional strategy provides a holistic view of treatment outcomes, enabling more informed and adaptive therapeutic decisions.

### Imaging evaluation

Imaging techniques—such as mammography, ultrasound, and MRI—are invaluable for monitoring TPBC treatment response. By providing precise measurements of tumor size and morphology, these techniques support timely treatment adjustments. Imaging allows healthcare providers to visualize tumor dynamics directly, enabling evidence-based decisions in breast cancer management.

### Biomarker analysis

Circulating biomarkers, such as cancer antigen 15-3 (CA 15-3), circulating tumor DNA (ctDNA), and circulating tumor cells (CTCs), offer valuable insights into the effectiveness of therapy. Tracking changes in CTCs and ctDNA provides a window into tumor burden and genomic shifts, helping predict response, identify potential resistance mechanisms, and tailor treatments accordingly (54–56). For example, ctDNA levels can indicate early signs of treatment response or resistance, while fluctuations in CTC counts can reflect changes in disease progression or remission status.

### Quality of life evaluation

Evaluating quality of life through patient-reported outcome measures (PROMs) offers a comprehensive view of treatment impact, addressing physical, emotional, and social well-being. PROMs ensure that treatment strategies extend beyond survival to focus on maintaining or improving quality of life. Patient feedback helps guide treatment adjustments, reducing side effects and enhancing adherence (57).

### Addressing disease progression or treatment failure

When treatment fails or disease progresses, reassessment with a focus on molecular profiling is essential. This involves analyzing biological changes to guide subsequent therapies. Proteomic analyses may detect specific molecular alterations, enabling the development of advanced SERMs, SERDs, and AIs to more effectively disrupt estrogen signaling and counteract resistance (58, 59). For example, mass spectrometry-based proteomics can identify signaling pathways that drive resistance, providing new targets for intervention in endocrine-responsive breast cancer. By understanding these molecular changes, personalized therapies can be designed to improve treatment outcomes and reduce the likelihood of further resistance (60–62).

### Understanding the tumor microenvironment

The tumor microenvironment (TME) plays a critical role in TPBC progression, as it includes not only tumor cells but also stromal cells (like adipocytes and immune cells) that can support or inhibit growth. Hormonal signals within the TME, particularly estrogen production and metabolism, contribute to cancer cell proliferation. Targeting these signals offers a novel approach, disrupting the TME to inhibit tumor growth and open new intervention pathways (63).

### Cross-pathway signal integration

Integrating hormone receptor signaling with survival pathways like PI3K/Akt/mTOR presents promising therapeutic opportunities. TPBC progression often activates these pathways, supporting cell survival and proliferation. Targeting convergence points across these pathways can disrupt tumor growth mechanisms and address the complexity of TPBC. By focusing on interwoven signaling networks, these therapies offer a sustainable path to managing cancer progression and overcoming resistance (64, 65).

### Strategies for combining therapies

Combining endocrine therapy, chemotherapy, targeted therapies (such as HER2 and CDK4/6 inhibitors), and immunotherapy enhances treatment efficacy in TPBC. A promising approach involves pairing immune checkpoint inhibitors (ICIs) with HER2-targeted therapies. Clinical studies have investigated combinations such as Atezolizumab (a PD-L1 inhibitor) with Trastuzumab Emtansine (T-DM1), showing that this combination may improve progression-free survival in advanced HER2-positive breast cancer by enhancing immune-mediated tumor destruction (41). Additionally, although primarily focused on triple-negative breast cancer (TNBC), studies with Pembrolizumab (a PD-1 inhibitor) and Atezolizumab in combination with chemotherapy have demonstrated the potential of ICIs to boost treatment efficacy and provide a rationale for using similar strategies in HER2-positive cancers (42, 43).

Furthermore, combining ICIs with adoptive T-cell therapies, like CAR-T cells engineered to target HER2, represents an innovative approach that may further improve outcomes by directly targeting tumor cells while also modulating the immune response. By leveraging the strengths of each treatment modality, these combinations create a synergistic effect: targeted therapies address cancer cell-specific mechanisms, while immunotherapy mobilizes the immune system. Ongoing research will clarify the most effective combinations and sequences, allowing responses to be optimized and side effects managed. This personalized strategy aligns with precision medicine by focusing on specific disease characteristics for each patient (66).

### Combining targeted therapy with endocrine treatments

Combining PARP inhibitors and CDK4/6 inhibitors with endocrine therapy is a leading-edge approach for managing TPBC, particularly for patients with BRCA mutations or other genetic predispositions. PARP inhibitors enhance endocrine therapy’s impact, especially effective in BRCA-mutant cancers, while CDK4/6 inhibitors prevent tumor cell proliferation by impeding cell division (67). Together, these therapies reduce tumor growth and mitigate resistance encountered with endocrine therapy alone. This personalized approach tailors treatment to the tumor’s molecular profile, maximizing efficacy and minimizing adverse effects. Such customized interventions improve outcomes and support quality of life, aligning with the goals of precision medicine by adapting to each tumor’s unique characteristics (38). In the MONARCH 2 trial, abemaciclib combined with fulvestrant yielded a PFS increase to 16.4 months compared to 9.3 months with fulvestrant alone, supporting CDK4/6 inhibitors’ use in managing hormone receptor-positive breast cancers. For TPBC, this indicates a potential strategy to mitigate resistance​.

### Application of molecular markers

Molecular markers are essential for precise treatment decisions in breast cancer management. These markers predict endocrine therapy response and identify potential resistance, enabling proactive treatment adjustments. Monitoring changes in ER, PR, HER2 expression, and genetic variations like ESR1 mutations provides insights into treatment response and resistance mechanisms (8, 68).

### Personalization of treatment choices

Personalized treatment utilizes advanced sequencing technology and bioinformatics to identify molecular markers in tumors, predicting response to specific therapies. By tailoring treatments based on gene mutations, expression patterns, and protein functions, this approach ensures each patient receives the most effective and targeted therapy, embodying the core principles of personalized oncology (69–71).

### Comprehensive consideration of patient characteristics

Personalized treatment for TPBC (Triple Positive Breast Cancer) goes beyond molecular profiling. It includes patient-specific factors such as genetic predisposition, unique tumor characteristics, lifestyle, and personal values. Integrating this information ensures that treatment plans are clinically effective and aligned with individual needs. This holistic approach tailors interventions to the patient’s biological and personal context, supporting the development of successful and sustainable strategies (72–74).

### Establishing an interdisciplinary care team

An interdisciplinary care team is vital for comprehensive TPBC management. This team, including oncologists, surgeons, radiologists, geneticists, bioinformaticians, genetic counselors, psychologists, and nutritionists, brings together diverse expertise to address multiple aspects of TPBC. Genetic counselors, in particular, help address hereditary cancer concerns, forming a solid support foundation for holistic care. This collaborative model integrates the latest advancements in cancer treatment, ensuring that patient care plans include promising new therapies. It not only enhances treatment effectiveness but also improves satisfaction and adherence, leading to better outcomes and quality of life (75–77).

### Patient participation in treatment decisions

Patient engagement in treatment decisions is essential for patient-centered care. Open communication and educational resources empower patients to make informed choices aligned with their health goals and lifestyle. This empowerment ensures that decisions are both medically sound and reflect the patient’s values and quality-of-life goals (78, 79). Patient advocacy groups and support networks play a crucial role by offering platforms for information sharing, helping patients navigate complex treatment options. Involving patients in decision-making improves adherence, as patients who understand the rationale behind their choices are more likely to commit to recommended therapies. This collaboration not only empowers patients but also fosters trust with healthcare providers, enhancing treatment effectiveness (80, 81).

## Conclusion

In conclusion, triple-positive breast cancer (TPBC) requires a highly personalized and multifaceted approach due to the complex interplay among estrogen, progesterone, and HER2 receptors. Tailored treatment strategies that integrate endocrine therapies, HER2-targeted agents, and novel immunotherapeutic approaches offer promising outcomes by addressing specific tumor characteristics and resistance mechanisms. Molecular profiling, including the analysis of signaling pathways and tumor microenvironment factors, has emerged as a crucial tool for optimizing therapy selection, as it allows clinicians to target multiple pathways simultaneously and enhance treatment efficacy.

The development of advanced proteomic and genomic biomarkers has further enabled the refinement of therapeutic interventions, improving the management of resistance and offering new targets for intervention. Future research should focus on enhancing combination therapy protocols, identifying predictive biomarkers, and refining patient selection criteria for immunotherapy to maximize therapeutic benefits. As precision medicine continues to evolve, these strategies will play an essential role in improving both survival and quality of life for TPBC patients, paving the way for more effective, individualized cancer care.
